# Physicochemical Stability of Hospital Parenteral Nutrition Solutions: Effect of Changes in Composition and Storage Protocol

**DOI:** 10.3390/pharmaceutics16050572

**Published:** 2024-04-23

**Authors:** Luis Otero-Millán, Brais Bea-Mascato, Jose Luis Legido Soto, Noemi Martínez-López-De-Castro, Natividad Lago-Rivero

**Affiliations:** 1 Pharmacy Department, University Hospital Complex of Vigo, 36312 Vigo, Spain; 2 NeumoVigo I+i Research Group, Galicia Sur Health Research Institute (IIS Galicia Sur), SERGAS-UVIGO, 36312 Vigo, Spain; 3 Innovation in Clinical Pharmacy Research Group (i-FARMA-Vigo), Galicia Sur Health Research Institute (IIS Galicia Sur), SERGAS-UVIGO, 36312 Vigo, Spain; 4 Applied Physic Department, Faculty of Sciences, University of Vigo, 36310 Vigo, Spain

**Keywords:** parenteral nutrition, physicochemical stability, critical care

## Abstract

(1) Background: Parenteral nutrition (PN) is a technique used for the administration of nutrients to patients for whom traditional routes cannot be used. It is performed using solutions with extremely complex compositions, which can give rise to a large number of interactions. These interactions can impact their stability and put the patient’s life at risk. The aim of this study is to determine how changes in composition and storage protocol affect the stability of NP solutions. (2) Methods: Twenty-three samples were prepared according to routine clinical practice, with modifications to the concentration of some components. The samples were stored at room temperature (RT) and refrigerated (4 °C). Measurements of the droplet diameter, pH, density and viscosity were performed for both storage protocols on days 1, 3, 10 and 14. (3) Results: The samples with the lowest concentration of lipids (PN13-17) and proteins (PN18-22) showed a larger droplet diameter than the rest of the samples throughout the experiments. The USP limits were exceeded for some of the measurements of these sample groups. The pH density and viscosity remained relatively constant under the conditions studied. (4) Conclusions: The PN samples were considered stable and safe for administration under real-world conditions, but the samples with the lowest concentrations of lipids and proteins showed a tendency towards emulsion instability.

## 1. Introduction

Parenteral nutrition (PN) is indicated for patients who cannot meet their nutritional requirements by oral or enteral route, either because their digestive system is not capable of the normal digestion and absorption of nutrients, or because it is necessary to keep the digestive tract at rest [1]. The enteral route is always the route of choice, as it is more physiological, has a lower risk of complications (lower risk of infections) and is also less costly [2]. Therefore, parenteral nutrition should be used when the enteral route cannot be used safely. Additionally, when oral or enteral tolerance is limited or nutritional needs are high, mixed nutrition can be used, i.e., the simultaneous use of parenteral and enteral nutrition [3].

The composition of a PN can vary depending on the specific needs of the patient, but generally includes a combination of macronutrients (carbohydrates, proteins and lipids) and micronutrients (vitamins, minerals and trace elements) [4]. Carbohydrates are provided in the form of glucose to provide energy, while proteins are provided in the form of amino acids for protein synthesis and to repair and maintain body tissues. Lipids are provided in the form of lipid emulsions to provide essential fatty acids and additional calories. In terms of micronutrients, a PN must contain an adequate amount of vitamins and minerals to avoid nutritional deficiencies. Trace elements, such as chromium, copper, iron, manganese, iodine, selenium and zinc, are also included in a PN in adequate amounts [5,6].

Focusing on the fact that we are dealing with a lipid emulsion, this turns out to be the first problem. Fat droplets are made up of non-polar molecules that are subject to strong Van der Waals forces, causing attraction, melting of the droplets, coalescence and the final separation of the emulsion phases. To prevent this from happening, the addition of amphipathic surfactants that cause the electrostatic repulsion of the droplets is necessary. In lipid emulsions used in PN mixtures, the fat droplets are coated with a thin layer of phospholipids (lecithin) that create a negative surface charge that causes this repulsion [7,8].

Lipid emulsions possess critical size characteristics, including the mean droplet diameter (MDD) and the range of droplet sizes distributed around this mean [9]. Notably, the fat droplets in the larger-diameter tail of the droplet size distribution play a crucial role in infusion safety, especially when their size exceeds 5 µm. The United States Pharmacopeia (USP), in chapter 729 [10], outlines the methods used to determine the median droplet diameter (MDD) and to assess the distribution of large-diameter droplets in lipid emulsions. These two regions of the droplet size distribution (mean droplet size and large-diameter tail) must adhere to specified limits [11]. Method I sets the upper limit for the average droplet size at 0.5 microns, while Method II evaluates the percentage of fat volume residing in droplets larger than 5 µm in the dispersed phase (referred to as PFAT5), and should not exceed 0.05% [12,13,14].

Incompatibilities can also occur due to the interaction of salts that are commonly added to PNs to provide electrolytes. Probably the most notorious incompatibility, and the one that has always been a major concern in the development of PN solutions, is that which can occur between calcium (Ca) and phosphorous (P) salts. The use of organic salts for both elements improves compatibility and reduces the risk of precipitation [15].

In addition to Ca-P precipitation, other studies have indicated that certain trace elements may interact with other nutrients to form precipitates. These elements could interact with proteins through redox reactions, forming high-affinity complexes. The amino acids in PNs, mainly sulfur-containing amino acids (cysteine and methionine), are affected by such reactions with elements, such as zinc, copper, iron and selenium [16].

The amount of macronutrients in a PN is adjusted according to the individual needs of each patient. At the hospital level, there are many situations in which changes in the composition of a PN are required that may affect its stability. Patients admitted to the hematology department after a hematopoietic transplantation may suffer from intestinal complications, with high water losses, mostly associated with electrolyte losses. For these reasons, it is necessary to increase the supply of these components (calcium, magnesium and potassium) in the PN. Patients admitted to critical care units and patients with heart disease are subject to volume restrictions, so the concentration and osmolarity of the PN increases. Finally, premature newborns have higher Ca requirements per kg of body weight than adults [17,18,19].

All these situations cause the approachment of the established composition–stability limits and compromise the safety of therapy for these patients. The administration of unstable PNs can have fatal repercussions for patients. Their destabilization, leading to the formation of large droplets, can lead to obstruction of the microvasculature, causing embolism [20,21,22,23,24].

In this study, we performed a comprehensive evaluation of the stability of different PN compositions.

## 2. Materials and Methods

### 2.1. Sample Preparation and Storage

For the evaluations made in this study, 23 parenteral nutrition stock solutions were prepared following routine clinical practice (Table 1). Sample 1 contained a standard formulation, with the macronutrient requirements used in hospital clinical practice. In this sample, a standard carbohydrate/lipid kilocalorie ratio of 60/40 and a non-protein kilocalorie/nitrogen gram ratio of 125 were calculated. From this baseline formulation, one of the elements was modified in increasing concentrations in the different groups of the subsequent solutions.

The processing procedure followed the standards and procedures concerning the cleaning and disinfection of the area, the use of aseptic techniques, the use of laminar flow cabinets (LFC) and the evaluation of the finished product. The latest Spanish consensus on the preparation of PN mixtures drawn up by the Working Group on Artificial Nutrition Pharmacy of the Spanish Society of Parenteral and Enteral Nutrition (SENPE) in 2008 was followed for the preparation [4].

A 500 mL stock sample was prepared, which was then separated into 250 mL for storage at room temperature (RT) and 250 mL for storage in a refrigerator (4 °C). The sample was redosed for storage in 50 mL polypropylene syringes (air free, luer-lock cap) from the original EVA bag and were stored for 14 days. For the measurements, the amount needed for the analyses was withdrawn from the syringe. Throughout the process, precautions were taken to protect the samples from light. Sterile materials were constantly used to avoid microbiological contamination of the samples. Furthermore, the preparation was performed in a sterile environment (laminar flow cabinets).

### 2.2. Study of Droplet Diameter

To assess the formation of droplets of different diameters, and to determine their distribution in the different samples, aliquots were independently introduced into a Saturn DigiSizer 5205^®^ (Micromeritics Inc., Norcross, GA, USA), which uses dynamic light scattering (DDL).

The measurements were performed on days 1, 3, 10 and 14, both in the samples kept at room temperature and at 4 °C (except on day 1, when only a single measurement was carried out at 4 °C). All the measurements were performed according to standard laboratory practice and the specific instructions of the equipment manufacturer. Initially, the stock solutions were gently shaken for homogenization after storage. A total of 5 mL of each stock solution was removed and was allowed to circulate for 1 min in the equipment until it was determined that the clogging value was stable (variation of less than 0.2%). The pumping rate used for the measurement was 12 L/min.

### 2.3. Density, Viscosity and pH Variation

The density measurements were conducted using an Anton Paar DMA4500^®^ vibrating tube, mechanical oscillation density meter (Graz, Austria). The temperature of the density meter was regulated through two integrated PT100 probes within the device itself, and system calibration was executed using standard fluids [25,26].

For the viscosity determination, we employed an Anton Paar AMV 200 viscometer^®^ (Graz, Austria). A constant test temperature was maintained using a PolyScience circulation bath (Cham, Suiza). Calibration was accomplished using standard liquids specific to each capillary [26,27].

The density and viscosity measurements were both conducted at room temperature (RT) and at 4 °C on days 1, 3, 10 and 14. To simplify the measurement protocol, measurements were only performed on samples with variations in lipid concentration. Substantial changes were not expected in the other samples. Every measurement represents the average of six viscosity determinations. For the density, only a single measurement was taken on day 0. The dynamic viscosity was calculated by multiplying the kinematic viscosity by the corresponding density.

The pH values of the examined mixtures were assessed using a Crison pH meter model Basic 20+^®^ (Barcelona, Spain), following the methodologies highlighted by Casas et al. and Carretero et al. [28,29]. In order to determine the main compounds influencing the pH of the solutions, pH measurements were carried out on all the samples. These measurements were carried out on day 1 only (samples at 4 °C). The measurements were executed while maintaining a controlled temperature of 25 °C. Approximately 8–10 mL of each individual sample was meticulously dispensed into 15 mL polypropylene centrifuge tubes. Subsequently, the loaded tubes were situated on a designated rack within the temperature-controlled bath, attaining the pre-set temperature of 25 °C. The measurement process for each sample was rigorously conducted in triplicate.

### 2.4. Visual Controls

After the preparation and on each day of analysis, a macroscopic examination was conducted on the bags to assess for phase separation, particle appearance or any alterations in the color of the solution.

### 2.5. Statistical Analysis

For the droplet size analysis, the data are presented using the median with the standard deviation. For the remaining experiments, the graphs illustrate the mean of the various replicates conducted during this study, whenever replicates were available.

Analyses were performed to evaluate the changes in droplet size distribution due to the different compositions, and were calculated for each sample and the different storage temperature. These analyses assessed the influence of time, temperature and composition on stability. Initially, to assess the impact of composition, a comparison was conducted between the PNBASE sample and the remaining PNx samples, all on the same day of measurement and under identical storage conditions (e.g., PNBASE on day 14 in ambient conditions versus PN22 on day 14 in ambient conditions).

Further analyses compared the differences due to the different composition of the samples at each time point and storage temperature (groups). To determine if there were significant differences among the groups, a Kruskal–Wallis test was initially used, where a *p*-value < 0.05 was considered as a significant result. When this test yielded positive results (*p*-value < 0.05), a post hoc analysis was followed by a pairwise comparison using the Wilcoxon test. A Benjamini–Hochberg multi-test correction (BH/FDR) was applied to the *p*-values of the Wilcoxon test, and an adjusted *p*-value (p.adj) < 0.05 was considered significant. These values are indicated in the figures with “*”: *, p.adj < 0.05; **, p.adj < 0.001; ***, p.adj < 0.0001; ****, p.adj < 0.00001. Moreover, in order to analyze the influence of the storage temperature on droplet size, each sample at RT was compared with its corresponding sample at 4 °C.

For statistical analyses and visualization, R (v4.2.2)^®^, Rstudio^®^ (v4.9.4) and the R packages ggplot2 (v 3.4.2)^®^, ggpubr (v0.6.0)^®^, tidyverse (v2.0.0)^®^ and rstatix (v0.7.2)^®^ were used. Due to the low number of samples in certain experiments, and the fact that the distribution of the data in the droplet sizing experiments did not follow a normal distribution, non-parametric statistics were used.

## 3. Results

### 3.1. Droplet Diameter

**Figure 1:**
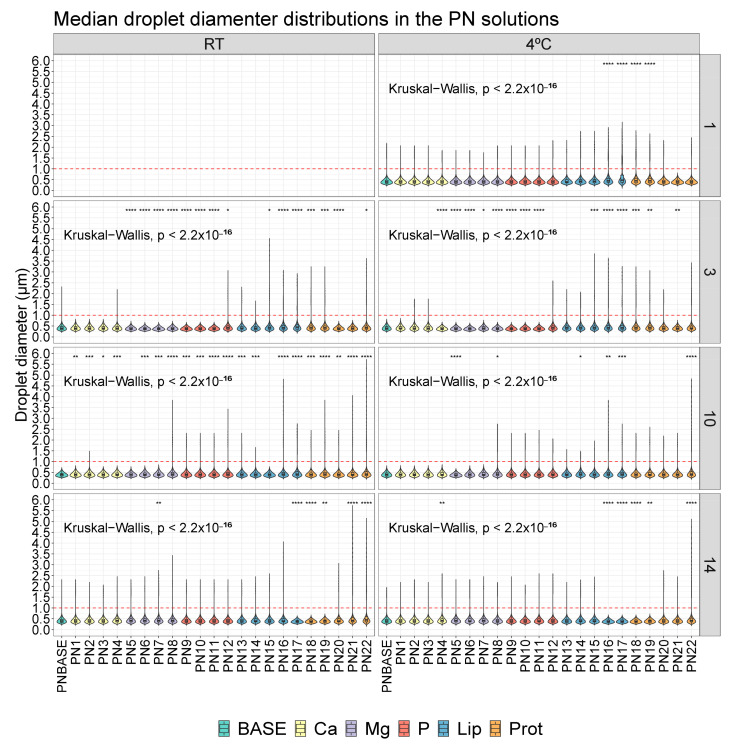
Distribution of droplet diameter in different parenteral nutrition (PN) solutions on different days (1, 3, 10 and 14) and temperatures (RT, 4 °C). RT: room temperature; Base: sample base; Ca: calcium; Mg: magnesium; P: phosphorus; Lip: lipids; Prot: protein. *: p.adj < 0.05; **: p.adj < 0.001; ***: p.adj < 0.0001; ****: p.adj < 0.00001.

A comparison was made between each PN1-PN22 sample and the PNBASE for the same day of measurement and under the same form of storage. Significant differences were observed in some samples (Figure 1), especially in the samples where we modified the concentration of lipids and proteins (PN13-PN22 samples).

**Figure 2:**
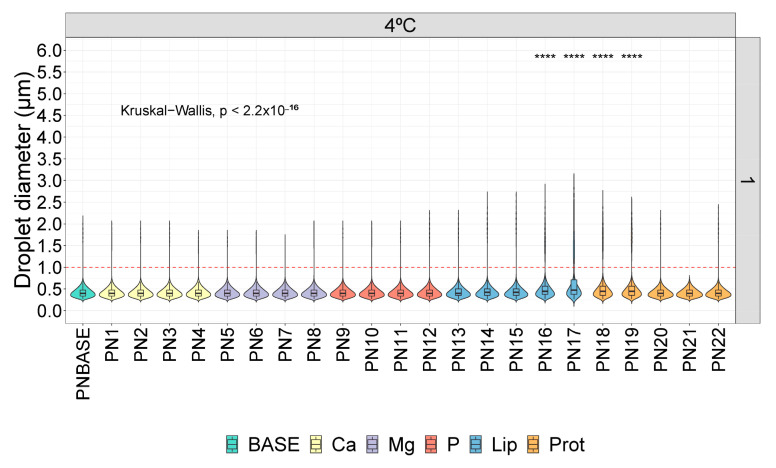
Distribution of droplet diameter in different parenteral nutrition (PN) solutions on day 1 and at 4 °C. Base: sample base; Ca: calcium; Mg: magnesium; P: phosphorus; Lip: lipids; Prot: protein; Lip: lipids. ****: p.adj < 0.00001.

Considering the baseline measurements on day 1 (Figure 2), statistically significant differences were observed in the following samples: PN16 and PN17, which are samples with decreasing lipid concentration; and PN18 and PN19, which are the first samples with a decreasing protein concentration.

**Figure 3:**
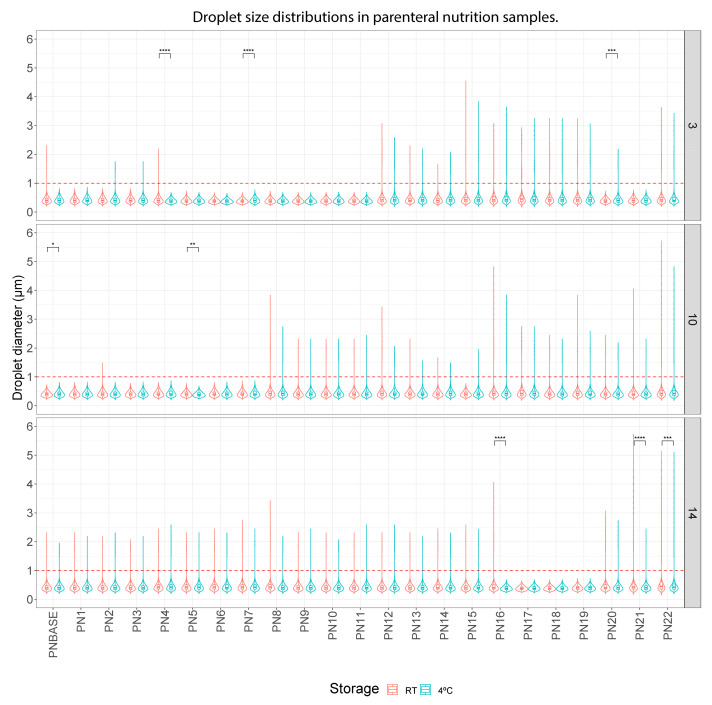
Distribution of droplet diameter in different parenteral nutrition (PN) solutions on different days (3, 10 and 14). Storage temperature comparison was RT vs. 4 °C. RT: room temperature. *: p.adj < 0.05; **: p.adj < 0.001; ***: p.adj < 0.0001; ****: p.adj < 0.00001.

On the other hand, Figure 3 shows the analysis performed to evaluate the impact of storage temperature. Overall, no relevant differences were observed in the droplet size of the PNBASE–PN22 samples according to their different storage protocols on any day of analysis (just some statistically significant differences in some of the samples).

**Figure 4:**
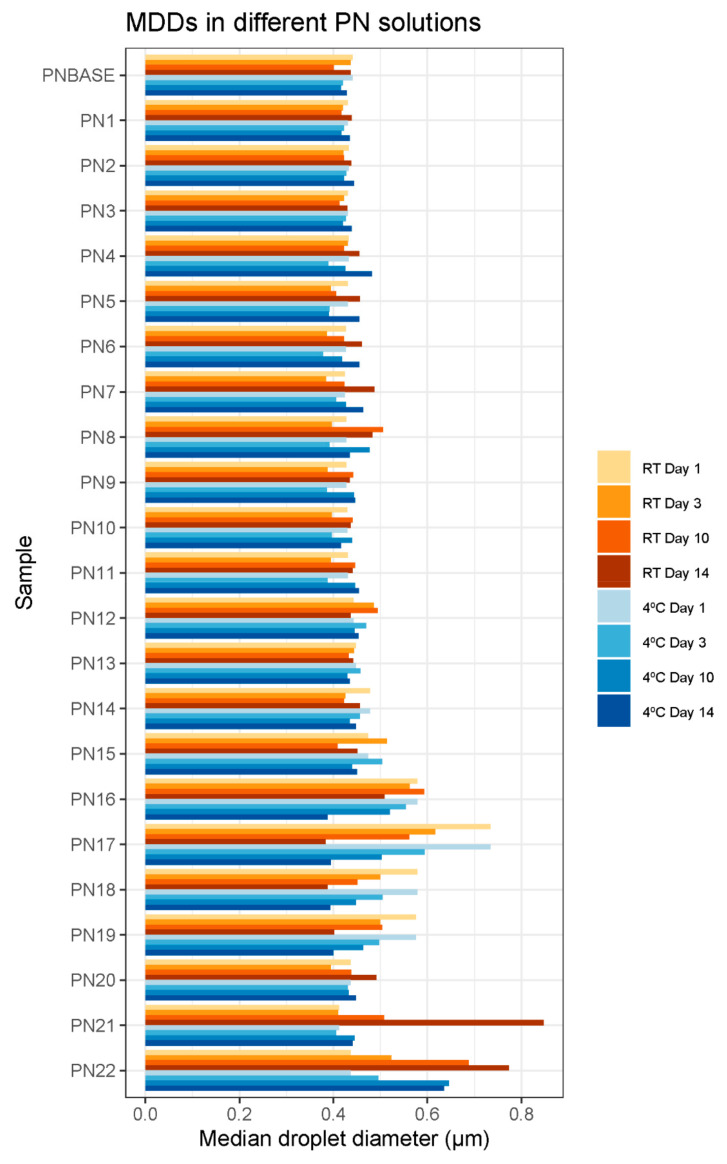
MDDs from samples PNBASE-PN22 for all days of analysis (1, 3, 10 and 14) and both storage conditions (RT: room temperature; 4 °C: refrigerator).

**Figure 5:**
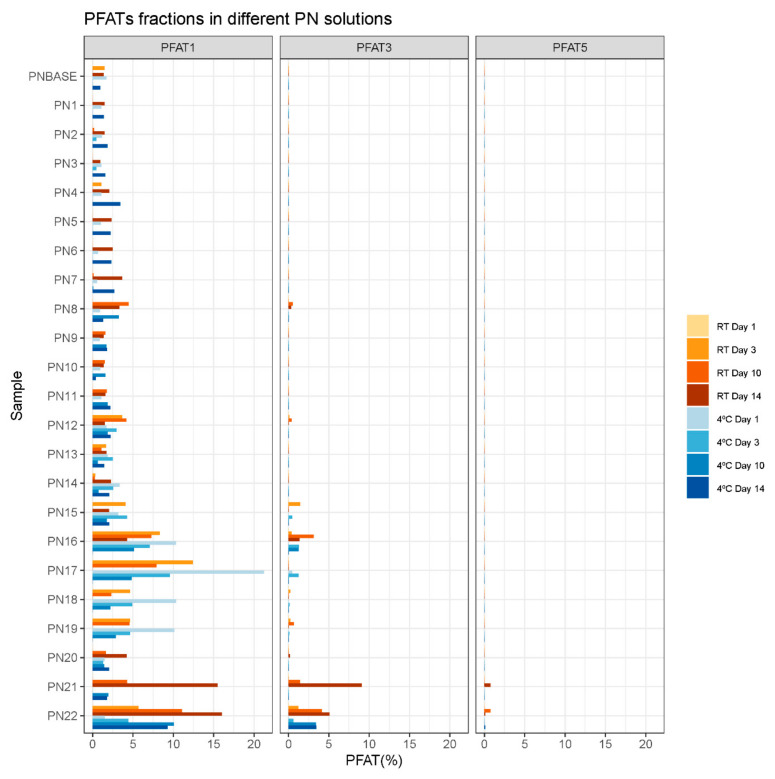
PFATs (1,3 and 5) from samples PNBASE-PN22 for all days of analysis (1, 3, 10 and 14) and both storage conditions (RT: room temperature; 4 °C: refrigerator).

The following parameters were calculated from the total diameter distribution of each sample: total median droplet diameter (MDD) and percentage of large droplets (PFAT1, PFAT3 and PFAT5) (Figure 4 and Figure 5, Tables S1–S5).

The MDD did not show large variations among the different samples prepared in this study, or among the storage protocols or time. An increase was only observed in samples PN8, PN15, PN16, PN17, PN18, PN19, PN21 and PN22, where the USP limit of 0.5 µm was exceeded.

The PFATs increased gradually as both lipid (PN13-17) and protein (PN18-22) concentrations decreased. This trend is especially visible in PFAT1, where much higher percentages of these groups were observed. Regarding PFAT3, it was only calculated for the lipid and protein sample groups (except for residual measurements of the other groups), and PFAT5 was only calculated for the protein group (Figure 5).

The complete data are shown in the Supplementary Materials. The supplementary tables contain some missing values, because no droplets above 1 μm were detected in any of the samples (Tables S1–S5).

In terms of compliance with USP parameters, the MDD limit of 0.5 µm was exceeded by several measurements, although only in the lipid and protein sample groups. Another exception was the PN8 sample, where this limit was slightly exceeded by a single measurement (MDD = 0.506 μm ± 0.003).

The PFAT5 limit (>0.05%) was exceeded by only four measurements after 10 or 14 days of storage in the samples with the lowest protein concentration (PN21 and PN22), which would be the only samples rejected as they simultaneously met both parameters. The rest of the measurements would comply with the established limits.

### 3.2. pH

**Figure 6:**
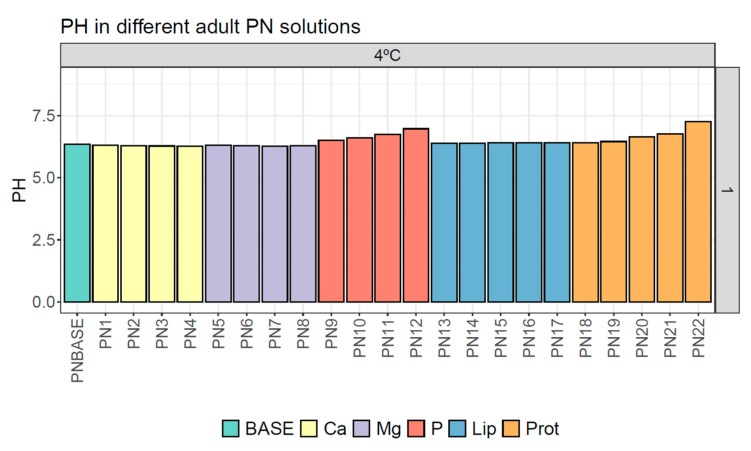
PH evaluation of different parenteral nutrition (PN) solutions (day 1, 4 °C).

It was observed that the pH values remained stable in most of the samples. A slight, non-significant increase in pH was observed in the samples where the phosphorus (PN9-PN12) and proteins (PN18-PN22) concentrations were altered (Figure 6).

### 3.3. Viscosity and Density

It was observed that the viscosity did not vary significantly with time or different storage temperatures (Table 2). However, a decreasing trend in viscosity was observed with a progressively decreasing lipid concentration of the sample.

No differences were observed in the density values (Table 3). The samples remained stable throughout the study period and according to the different preservation forms.

## 4. Discussion

Our study aims to evaluate the stability of the various physicochemical properties characteristic of parenteral nutrition solutions used in clinical practice. To this end, different samples were prepared by altering their composition and they were subjected to different storage protocols.

Firstly, storage time and temperature are factors related to the instability of lipid emulsions, leading to phase separation and, ultimately, to emulsion breakage. Storage time seems to be a conditioning factor for stability in our analyses. Although disturbances (for example, increases in droplet size or increases in USP limits) were observed on both day 1 and day 14, they appear to have increased in frequency over time (only lipid droplets larger than 5 microns were detected after 10 or 14 days of study) (Figure 4 and Figure 5, Tables S1–S5). However, temperature did not seem to affect stability, as the samples stored at room temperature and at 4 °C did not show a substantial difference in the frequency of disturbances (Figure 3). We find the data less robust than, for example, those obtained for the changes in lipid and protein concentrations, as explained below.

In this study, some samples exceeded the limits established by the USP. This deviation was observed mainly in two groups of samples: those where the lipid concentration was modified (PN13-PN17), and those where the protein concentration was modified (PN18-PN22). The limit was also slightly exceeded by a single measurement of the PN8 sample (MDD = 0.506 μm ± 0.003) (Figure 4).

Concerning the MDD, we observed that the 0.5 µm limit was exceeded by the groups mentioned above. Alterations in the total droplet diameter occurred as early as day 1 for samples PN14, PN16, PN17, PN18 and PN19. After 10 and 14 days, this limit was also exceeded by samples PN15, PN19, PN20, PN21 and PN22. An MDD greater than 0.5 µm is not sufficient to exclude a PN, which is why the USP uses this parameter together with PFAT5. The PFAT5 value was met by virtually all the measurements performed. Looking at the data shown in Tables S1–S5, the percentage of droplets corresponding to the largest size distribution (excluding the first peak) does not usually exceed 5%.

We completed examination of the MDD data with sub-analyses of the fraction of droplets larger than 1 µm (Figure 5). This fraction of larger droplets is sometimes under-represented in the total diameter distribution, so these data were analyzed with caution when drawing conclusions. However, they can be used to observe trends that, complemented with the rest of the data obtained, can help us to characterize critical limits in stability. In this respect, the PFAT1 results show a trend towards an increasing droplet diameter in the samples where we modified the concentration of lipids and proteins (PN13-PN22 samples) compared to the other samples. For the PN13-PN22 samples, a PFAT1 value of 0–21.25% was observed, compared to the rest of the samples, where it varied from 0 to 4.46% (Tables S1–S5). More specifically, for the fractions larger than 3 µm (PFAT3) and larger than 5 µm (PFAT5), the difference was more pronounced. The PFAT3 value was only calculated for samples PN14-PN22, PN8 (higher magnesium concentration) and PN12 (higher phosphorus concentration). In the rest of the samples, the distribution was always smaller than 3 µm.

PFAT5 is the other control parameter proposed by the USP, which proposes a limit of PFAT5 < 0.05%. In our study, droplets larger than 5 µm were only detected in two samples, PN21 and PN22, in their measurements on days 10 and 14 (PN21_RT_D14, PN22_RT_D10, PN22_RT_D14 and PN22_4 °C_D14). For all of them, the limit of 0.05% was exceeded. It should be noted that these are the samples with a lower protein concentration. According to the parameters established by the USP, only these two samples should be discarded 10–14 days after preparation (Figure 5).

The PFAT5 parameter is widely used in PN stability studies. Driscoll et al. [8,12] described possible scenarios of changes in the PFAT5 value over time. In another work by Driscoll et al. [14], PFAT5 values greater than 0.4% correlated with unstable emulsions where phase separation was visible. In PN21_RT_D14 and PN22_RT_D10, the PFAT5 value was 0.746% and 0.736%, respectively (Figure 4). No visible phase separation was ever observed in these samples. Phase separation was also never reported by the external measurement personnel. However, with a more thorough visual inspection protocol based on light lamps under black/white backgrounds, as performed in other studies, perhaps other types of alterations could be observed.

Our results appear to be consistent with those in the literature, where it has been described that low lipid and protein concentrations are associated with a lower stability of PN samples. Along this line, Driscoll et al. [30] extended an earlier work in order to extend the data on the limits of their stability in a wider range of parenteral formulations. Eight neonatal PNs of different macronutrient ratios were studied. A specialized amino acid formulation for neonates (TrophAmine 10%^®^) was used at concentrations of 1%, 1.5%, 3% and 4%. The eight formulations were prepared in triplicate (*n* = 24) and studied for 30 h at room temperature (25 ± 2 °C). The USP measurements for MDD and PFAT5 were performed with DDL and LE/SPOS, respectively. Regarding PFAT5, the PN samples with 1% and 1.5% protein had values above 0.05% (up to 0.50%) in most of the samples (68 out of 96, or 71% of the cases). The samples with amino acid concentrations of 3% and 4% showed a PFAT5 < 0.05% (up to 0.04%) in 100% of the cases (96/96). These results show a critical point with respect to the PFAT5 limits in USP chapter 729.

On the other hand, low lipid concentrations may also destabilize an emulsion. However, this conclusion, which has been widely used by different consensuses [4], is not supported by studies analyzing different lipid concentrations. A possible explanation for this destabilization could be the larger surface area of the droplets. At higher concentrations, the lipid droplets would be closer together, making the destabilizing action of cations more difficult. A lower concentration of lipids would mean a lower number of lipid globules. This would make them more likely to interact with other species, such as cations, which could disrupt the protective electrostatic layer formed by phospholipids. On the other hand, a higher number of droplets could also lead to a greater likelihood of collisions among them, and a corresponding melting and enlargement of the droplets. Given the complex composition of PN mixtures, a superposition of all these factors is to be expected, making it difficult to determine which of them plays a more relevant role. In our study, we observed a clear trend towards larger emulsions in samples with lower lipid concentrations. These observations require further study to confirm this hypothesis.

It should be noted that some differences in droplet diameter were found in samples where the fraction of droplets above 1 µm was not present on days 3 and 10. In this study, some technical limitations in the measurement of the lipid droplet size could have affected the results obtained. A strict adherence to equipment specifications was maintained, yet our inability to detect droplet fractions exceeding 1 µm may be attributed to inherent technical limitations. The “fog effect” phenomenon, observed in emulsions with a higher concentration of small droplets, results in significant radiation reflection, causing inaccuracies in larger particle population assessments. Despite these challenges, our findings align with those in the literature indicating a lack of information on larger droplets in analogous samples assessed using different methodologies [31,32].

We calculated PFAT5 from our lipid droplet size distribution data. This may represent another limitation of this study, since the USP recommends the use of the light obscuration or light extinction method for this type of calculation. The extrapolation and direct relationship of our data with the cut-off point of PFAT5 > 0.005, established by the USP, could be limited. In any case, we believe that its calculation is also interesting for observing a trend towards a possible growth in droplet size according to different compositions and storage conditions. This study expands the existing knowledge of PN stability, and the data obtained could be useful for further studies on this topic.

Another potential source of error arises from substance adherence to containers during sample storage and measurement. Gonyon et al. [33] investigated the relationship between emulsion droplet size changes and lipid adsorption to container materials in parenteral nutrition mixtures. Comparing glass bottles to ethyl vinyl acetate (EVA) plastic containers, they found significantly higher adsorption to plastic across their measurements. Samples in EVA containers showed a 75% PFAT5 reduction, while glass containers exhibited a marginal decrease. Our samples were stored in EVA bags, which may explain the absence of >1 µm droplet fractions. Further validation would require a study of samples stored in different containers with individualized analysis.

Additional research by Driscoll et al. [8,12] suggests that factors like agitation and air incorporation into bags may disrupt droplet size measurements. They attribute an initially elevated PFAT5 to increased air or bubbles in the initial measurements, a phenomenon that diminishes over the storage time.

It should also be noted that our data are limited to the use of the commercial formulations indicated in the Section 2. Other formulations may show different physicochemical behavior due to slight changes in composition.

The relationship between instability and high cation concentrations has been extensively studied in the literature [31,32,34,35]. Cations neutralize the negative charge of the phospholipid layer covering the surface of the droplets, increasing the probability of the aggregation and melting of the droplets. In our data, despite the high concentrations of calcium and magnesium in certain samples (PN1-PN8), no values typical of unstable samples were observed, in contrast to the groups of samples where we varied the protein and lipid concentrations.

Our study includes samples with a wide range of concentrations of the components used in the production of PNs. This generated samples with a wide range of CAN values. The CAN is a parameter historically related to lipid emulsion stability. Its value is the sum of the electrolyte concentrations, which may be involved in lipid aggregation. In our study, the values varied from 2020 mMol/L to 6020 mMol/L. The maximum value was reached in the samples where the calcium and magnesium concentrations were increased (PN1-PN8), as divalent cation concentrations have a greater influence on the CAN value. The rest of the samples had the same CAN value, excepting for slight variations. The samples with varying phosphorus concentrations (PN9-PN12) were accompanied by variations in sodium (Na). However, the influence of this cation on the CAN is minor, resulting in a concentration of no more than 2180 mMol/L. The most unstable groups of samples, where the lipids and proteins were modified, did not exceed 2020 mMol/L for the CAN value, so we have not found any relationship between a higher CAN value and a higher MDD, increases in the fraction of large droplets or a greater occurrence of the precipitate. Therefore, there is no relationship between higher CAN values and the instability of our PN mixtures.

The CAN calculation is simple, as it is only based on the concentration of cations in a solution. However, it does not consider other factors such as pH and protein concentration, which, as we have observed, play important roles when talking about stability [36]. Not taking other factors into account can underestimate or overestimate the stability of an emulsion. Due to this factor, the utilization and importance of the CAN in clinical settings have experienced a decline in recent years. In contrast, the parameters defined by the USP (MDD and PFAT5) have gained popularity. The latter demonstrates a more immediate association with lipid coalescence and aggregation, proving to be more effective predictors of instability in PN solutions. However, performing instant calculations of these parameters in routine clinical practice is, presently, impractical. As a result, the CAN, along with the calcium or phosphorus concentrations and macronutrients, continues to serve as the benchmark for anticipating aggregation and instability states in PN solutions. Along this line, we also included the calculation of osmolarity, as it is an important variable for the calculation of PN composition in a clinical setting. It is of importance to choose the route of administration of the PN (there are different osmolarity limits for the peripheral or central route). However, its impact on stability has not been studied in the literature and we have not assessed it either. Osmolarity includes the calculation of all ionic species and, theoretically, the anions in a solution are not involved in stability. Despite this, samples PN13-PN22 had the lowest osmolarity in our study and, as mentioned above, they are the samples where we detected the greatest signs of destabilization. Therefore, it could be interesting to evaluate the possible impact of low osmolarity on PNs in future studies. In summary, there is a need to establish new parameters capable of predicting PN stability and that are applicable to the daily operations of hospitals.

In our samples, we observed two groups of compositions where the pH changes were more pronounced. These groups include the samples where the concentration of phosphorus (PN9-PN12) or proteins (PN18-PN22) was modified (Figure 6). In a PN mixture, phosphate is one of the components that can dissociate and affect the pH of the solution. Proteins in PNs are added as amino acid solutions. Amino acids are amphoteric molecules, i.e., they can act both as acids and bases depending on the pH of the medium in which they are found.

For our pH results, the maximum value was measured in the sample with the highest concentration of glycerophosphate (PN12), with a pH around 7, and in PN22, with a pH of 7.26 (the sample without proteins). In the rest of the samples, the pH was between 6.3 and 6.4.

These results are in line with those observed in the literature [37]. In the studies consulted, no significant alterations in pH values were usually observed in relation to storage time or temperature. It is a parameter that remained relatively stable in most studies. If changes were observed, they usually led to a decrease in pH values after long storage times, as in the study by Janu et al. [35]. In another study published by Driscoll et al. [8], the pH values did not change substantially among the different samples or among the different measurement times, so it was not an important factor affecting stability. They also observed that once lipids are mixed with other additives, there is a reduction in pH due to the hypertonic glucose.

The pH among the solutions from different studies may have varied depending on the components used in the sample preparation. Differences in composition could lead to the appearance of species in the solutions with different behaviors and different pHs. One of the components that can have a major impact on this process is the protein source chosen. In our study, we used Aminoven Infant 10%^®^, which, according to its data sheet, has a pH between 5.5 and 6.0. Pediatric amino acid profiles have a higher branched-chain amino acid content, contain taurine and are more acidic, which may lead to greater sample instability compared to adult profiles [11,38,39].

Finally, the results obtained for density and viscosity did not allow us to observe trends in relation to stability (Table 2 and Table 3). The samples remained stable over time for both storage protocols. Only slight changes correlated with changes in the lipid concentrations of the samples were observed. These are expected and predictable.

## 5. Conclusions

The PN samples were considered stable and safe for administration under real-world conditions of use, since the USP specifications were met for most of the conditions tested, except for the use of low concentrations of lipids and proteins, where a tendency towards instability in the lipid emulsion was observed. On the other hand, high concentrations of cations (high CAN) did not show a correlation with the greater instability of the lipid emulsion.

The density and viscosity exhibited relatively consistent levels in the parenteral nutrition samples, with slight increases observed as the macronutrient concentrations increased. The pH of the samples also remained constant, with slight variations depending on the conditions of the storage protocol that were altered. Changes in the protein and phosphate concentrations caused an increase in pH values, but without repercussions on stability.

It is important to consider that the sample handling conditions for lipid droplet size analysis could affect the interpretation of the results, such as the analytical equipment or containers used for transport.

## Figures and Tables

**Table 1:** Details of modifications made to sample formulation used in this study. Calculation of CAN and OSM are included.

Sample	N (g/L)	Prot (g/L)	Gluc (g/L)	Lip (g/L)	Na (mMol/L)	K (mMol/L)	Mg (mMol/L)	Ca (mMol/L)	P (mMol/L)	OSM (mOsm/L)	CAN (mMol/L)
PN BASE	4.00	25.00	75.00	20.00	40.00	60.00	5.00	25.00	12.50	1030.00	2020.00
PN1	4.00	25.00	75.00	20.00	40.00	60.00	5.00	50.00	12.50	1130.00	3620.00
PN2	4.00	25.00	75.00	20.00	40.00	60.00	5.00	62.50	12.50	1180.00	4420.00
PN3	4.00	25.00	75.00	20.00	40.00	60.00	5.00	75.00	12.50	1230.00	5220.00
PN4	4.00	25.00	75.00	20.00	40.00	60.00	5.00	87.50	12.50	1280.00	6020.00
PN5	4.00	25.00	75.00	20.00	40.00	60.00	12.50	25.00	12.50	1060.00	2500.00
PN6	4.00	25.00	75.00	20.00	40.00	60.00	25.00	25.00	12.50	1110.00	3300.00
PN7	4.00	25.00	75.00	20.00	40.00	60.00	37.50	25.00	12.50	1160.00	4100.00
PN8	4.00	25.00	75.00	20.00	40.00	60.00	50.00	25.00	12.50	1210.00	4900.00
PN9	4.00	25.00	75.00	20.00	40.00	60.00	5.00	25.00	20.00	1043.50	2020.00
PN10	4.00	25.00	75.00	20.00	48.00	60.00	5.00	25.00	30.00	1101.50	2028.00
PN11	4.00	25.00	75.00	20.00	80.00	60.00	5.00	25.00	50.00	1217.50	2060.00
PN12	4.00	25.00	75.00	20.00	120.00	60.00	5.00	25.00	100.00	1507.50	2100.00
PN13	4.00	25.00	75.00	15.00	40.00	60.00	5.00	25.00	12.50	1015.00	2020.00
PN14	4.00	25.00	75.00	10.00	40.00	60.00	5.00	25.00	12.50	1000.00	2020.00
PN15	4.00	25.00	75.00	5.00	40.00	60.00	5.00	25.00	12.50	985.00	2020.00
PN16	4.00	25.00	75.00	2.50	40.00	60.00	5.00	25.00	12.50	977.50	2020.00
PN17	4.00	25.00	75.00	1.24	40.00	60.00	5.00	25.00	12.50	973.75	2020.00
PN18	3.50	21.87	75.00	20.00	40.00	60.00	5.00	25.00	12.50	995.63	2020.00
PN19	3.00	18.75	75.00	20.00	40.00	60.00	5.00	25.00	12.50	961.25	2020.00
PN20	2.50	15.62	75.00	20.00	40.00	60.00	5.00	25.00	12.50	926.88	2020.00
PN21	1.50	9.37	75.00	20.00	40.00	60.00	5.00	25.00	12.50	858.13	2020.00
PN22	0.00	0.00	75.00	20.00	40.00	60.00	5.00	25.00	12.50	755.00	2020.00

PNBASE-PN22: PN samples; N: nitrogen (Aminoven Infant 10% Fresenius Kabi^®^ Barcelona, Spain); Prot: protein; Gluc: glucose (Glucose 50% Grifols^®^ Barcelona, Spain); Lip: lipids (Lipoplus 20% Braun^®^, Melsungen, Germany); OSM: osmolarity; CAN: critical aggregation number, calculated according to cation concentration to analyze its relationship to stability (CAN = a + 64 b + 729 c, where a, b and c are the sum of the concentrations (mmol/L) of mono-, di- and trivalent cations, respectively). Other components used: Sodium Chloride 20% Braun^®^, Potassium Acetate 1M Braun^®^, sodium glycerophosphate (Glycophos Fresenius Kabi^®^), calcium gluconate (Suplecal Braun^®^), and Magnesium Sulfate 15% Genfarma^®^ Madrid, Spain. Vitamins: Vitalipid Fresenius Kabi^®^. Trace elements: Meinsol Oligo-zinc Fresenius Kabi^®^ and water for injection (Grifols^®^).

**Table 2:** Viscosity (MPa·s) of PN13-PN17 samples (temperature = 25 °C). All days of analysis (1, 3, 10 and 14) and both storage conditions (RT: room temperature, 4 °C: refrigerator).

Sample	Day 1	Day 3		Day 10		Day 14	
	4 °C	4 °C	RT	4 °C	RT	4 °C	RT
PN13	1.415 ± 0.004	1.402 ± 0.003	1.394 ± 0.003	1.399 ± 0.003	1.397 ± 0.004	1.411 ± 0.007	1.394 ± 0.004
PN14	1.365 ± 0.008	1.361 ± 0.005	1.368 ± 0.005	1.369 ± 0.004	1.365 ± 0.004	1.375 ± 0.003	1.387 ± 0.005
PN15	1.370 ± 0.004	1.344 ± 0.003	1.343 ± 0.004	1.347 ± 0.006	1.343 ± 0.004	1.357 ± 0.004	1.351 ± 0.004
PN16	1.343 ± 0.004	1.329 ± 0.003	1.321 ± 0.005	1.334 ± 0.005	1.356 ± 0.002	1.334 ± 0.005	1.331 ± 0.004
PN17	1.327 ± 0.005	1.316 ± 0.003	1.316 ± 0.003	1.320 ± 0.005	1.346 ± 0.003	1.320 ± 0.004	1.318 ± 0.003

**Table 3:** Density (g/cm^3^) of PN13-PN17 samples (temperature = 25 °C), for all days of analysis (1, 3, 10 and 14) and both storage conditions (RT: room temperature; 4 °C: refrigerator).

Sample	Day 1	Day 3		Day 10		Day 14	
	4 °C	4 °C	RT	4 °C	RT	4 °C	RT
PN13	1.045	1.049	1.045	1.049	1.045	1.045	1.045
PN14	1.044	1.044	1.044	1.044	1.044	1.044	1.044
PN15	1.045	1.045	1.045	1.045	1.045	1.045	1.045
PN16	1.045	1.045	1.045	1.045	1.045	1.046	1.045
PN17	1.045	1.045	1.045	1.045	1.045	1.045	1.045

## Data Availability

Data is contained within the article or Supplementary Material.

## Supplementary Materials

The following supporting information can be downloaded at: https://www.mdpi.com/article/10.3390/pharmaceutics16050572/s1, Table S1: Calculated droplet diameter parameters for increasing [calcium] samples (PNBASE, PN1-PN4), for all days of analysis (1, 3, 10 and 14) and both storage conditions (RT: room temperature; 4 °C: refrigerator); Table S2: Calculated droplet diameter parameters for increasing [magnesium] samples (PN5-PN8), for all days of analysis (1, 3, 10 and 14) and both storage conditions (RT: room temperature; 4 °C: refrigerator); Table S3: Calculated droplet diameter parameters for increasing [phosphorus] samples (PN9-PN12), for all days of analysis (1, 3, 10 and 14) and both storage conditions (RT: room temperature; 4 °C: refrigerator); Table S4: Calculated droplet diameter parameters for decreasing [lipid] samples (PN13-PN17), for all days of analysis (1, 3, 10 and 14) and both storage conditions (RT: room temperature; 4 °C: refrigerator); Table S5: Calculated droplet diameter parameters for decreasing [amino acid] samples (PN18-PN22), for all days of analysis (1, 3, 10 and 14) and both storage conditions (RT: room temperature; 4 °C: refrigerator).

## References

[1] Boullata J.I., Gilbert K., Sacks G., Labossiere R.J., Crill C., Goday P., Kumpf V.J., Mattox T.W., Plogsted S., Holcombe B. (2014). ASPEN clinical Guidelines: Parenteral Nutrition Ordering, Order Review, Compounding, Labeling, and Dispensing. JPEN J. Parenter. Enteral Nutr..

[2] Thibault R., Abbasoglu O., Ioannou E., Meija L., Ottens-Oussoren K., Pichard C., Rothenberg E., Rubin D., Siljamäki-Ojansuu U., Vaillant M.-F. (2021). ESPEN Guideline on Hospital Nutrition. Clin. Nutr..

[3] Boullata J.I., Berlana D., Pietka M., Klek S., Martindale R. (2020). Use of Intravenous Lipid Emulsions with Parenteral Nutrition: Practical Handling Aspects. JPEN J. Parenter. Enteral Nutr..

[4] Pera D.C., Peris M.C., Arévalo M.F., Muñoz P.G., Tutor M.J.M., Corrales G.P., Penín I.R., Polo A.V. (2009). Consenso Español Sobre la Preparación de Mezclas Nutrientes Parenterales. Farm. Hosp..

[5] Planas M. (2008). Conceptos Prácticos en Nutrición Enteral y Parenteral.

[6] Muñoz P.G., Zanuy M.V., Hernández Á.G., De Nutrición T. (2010). Nutrición Parenteral.

[7] Driscoll D.F., Nehne J., Peterss H., Klütsch K., Bistrian B.R., Niemann W. (2003). Physicochemical Stability of Intravenous Lipid Emulsions as All-in-One Admixtures Intended for the Very Young. Clin. Nutr..

[8] Driscoll D.F., Thoma A., Franke R., Klütsch K., Nehne J., Bistrian B.R. (2009). Lipid Globule Size in Total Nutrient Admixtures Prepared in Three-Chamber Plastic Bags. Am. J. Health Syst. Pharm..

[9] Otero-Millán L., Lago Rivero N., Blanco Rodicio A., García Beloso N., Legido Soto J.L., Piñeiro-Corrales G. (2021). Stability of Lipid Emulsion in Total Parenteral Nutrition: An Overview of Literature. Clin. Nutr. ESPEN.

[10] Rockville M.D. (2023). Globule Size Distribution in Lipid Injectable Emulsions. United States Pharmacopeia.

[11] Skouroliakou M., Matthaiou C., Chiou A., Panagiotakos D., Gounaris A., Nunn T., Andrikopoulos N. (2008). Physicochemical Stability of Parenteral Nutrition Supplied as All-in-One for Neonates. JPEN J. Parenter. Enteral Nutr..

[12] Driscoll D.F., Parikh M., Silvestri A.P., Klütsch K., Bistrian B.R., Nehne J. (2006). Establishing a Stability Window for Medium- and Long-Chain-Triglyceride Lipid-Based Total Nutrient Admixtures Using USP Standards. Am. J. Health-Syst. Pharm..

[13] Driscoll D.F., Giampietro K., Wichelhaus D.P., Peterss H., Nehne J., Niemann W., Bistrian B.R. (2001). Physico-Chemical Stability Assessments of Lipid Emulsions of Varying Oil Composition. Clin. Nutr..

[14] Driscoll D.F., Silvestri A.P., Nehne J., Klütsch K., Bistrian B.R., Niemann W. (2006). Physicochemical Stability of Highly Concentrated Total Nutrient Admixtures for Fluid-Restricted Patients. Am. J. Health-Syst. Pharm..

[15] Newton D.W., Driscoll D.F. (2008). Calcium and Phosphate Compatibility: Revisited Again. Am. J. Health-Syst. Pharm..

[16] Foinard A., Perez M., Barthélémy C., Lannoy D., Flamein F., Storme L., Addad A., Bout M.-A., Décaudin B., Odou P. (2016). In Vitro Assessment of Interaction Between Amino Acids and Copper in Neonatal Parenteral Nutrition. JPEN J. Parenter. Enteral Nutr..

[17] Mehta N.M., Skillman H.E., Irving S.Y., Coss-Bu J.A., Vermilyea S., Farrington E.A., McKeever L., Hall A.M., Goday P.S., Braunschweig C. (2017). Guidelines for the Provision and Assessment of Nutrition Support Therapy in the Pediatric Critically Ill Patient: Society of Critical Care Medicine and American Society for Parenteral and Enteral Nutrition. J. Parenter. Enter. Nutr..

[18] Mihatsch W.A., Braegger C., Bronsky J., Cai W., Campoy C., Carnielli V., Darmaun D., Desci T., Domellöf M., Embleton N. (2018). ESPGHAN/ESPEN/ESPR/CSPEN Guidelines on Pediatric Parenteral Nutrition. Clin. Nutr..

[19] Otero-Millán L., Bea-Mascato B., Legido Soto J.L., Martínez-López-De-Castro N., Lago-Rivero N. (2024). Evaluation of the Stability of Newborn Hospital Parenteral Nutrition Solutions. Pharmaceutics.

[20] Driscoll D.F., Ling P.-R., Quist W.C., Bistrian B.R. (2005). Pathological Consequences from the Infusion of Unstable Lipid Emulsion Admixtures in Guinea Pigs. Clin. Nutr..

[21] Driscoll D.F. (2007). Quality, stability and safety of lipid emulsions. Clin. Nutr..

[22] Gerard-Boncompain M., Claudel J.P., Gaussorgues P., Salord F., Sirodot M., Chevallier M., Robert D. (1992). Hepatic Cytolytic and Cholestatic Changes Related to a Change of Lipid Emulsions in Four Long-Term Parenteral Nutrition Patients with Short Bowel. J. Parenter. Enter. Nutr..

[23] Toce S.S., Keenan W.J. (1995). Lipid Intolerance in Newborns Is Associated with Hepatic Dysfunction but Not Infection. Arch. Pediatr. Adolesc. Med..

[24] Mckinnon B.T. (1996). FDA Safety Alert: Hazards of Precipitation Associated with Parenteral Nutrition. Nutr. Clin. Pract..

[25] Lago A., Rivas M.A., Legido J., Iglesias T.P. (2009). Study of Static Permittivity and Density of the Systems {(n-Nonane+monoglyme or Diglyme)} at Various Temperatures. J. Chem. Thermodyn..

[26] Pastoriza-Gallego M.J., Casanova C., Legido J.L., Piñeiro M.M. (2011). CuO in Water Nanofluid: Influence of Particle Size and Polydispersity on Volumetric Behaviour and Viscosity. Fluid Phase Equilibria.

[27] Pastoriza-Gallego M.J., Casanova C., Páramo R., Barbés B., Legido J.L., Piñeiro M.M. (2009). A Study on Stability and Thermophysical Properties (Density and Viscosity) of Al2O3 in Water Nanofluid. J. Appl. Phys..

[28] Carretero M.I., Pozo M., Legido J.L., Fernández-González M.V., Delgado R., Gómez I., Armijo F., Maraver F. (2014). Assessment of Three Spanish Clays for Their Use in Pelotherapy. Appl. Clay Sci..

[29] Casás L.M., Pozo M., Gómez C.P., Pozo E., Bessières L.D., Plantier F., Legido J.L. (2013). Thermal Behavior of Mixtures of Bentonitic Clay and Saline Solutions. Appl. Clay Sci..

[30] Driscoll D.F., Silvestri A.P., Bistrian B.R. (2010). Stability of MCT/LCT-Based Total Nutrient Admixtures for Neonatal Use over 30 Hours at Room Temperature: Applying Pharmacopeial Standards. JPEN J. Parenter. Enteral Nutr..

[31] Watrobska-Swietlikowska D., Szlagatys-Sidorkiewicz A., Łuszkiewicz K. (2014). Evaluation of Physical Stability of All in One Parenteral Admixtures for Pediatric Home Care with High Electrolytes Concentrations. Nutr. Hosp..

[32] Watrobska-Swietlikowska D., Szlagatys-Sidorkiewicz A., MacLoughlin R. (2018). The Presence of Inorganic Calcium in Pediatric Parenteral Admixtures. Nutr. Hosp..

[33] Gonyon T., Tomaso A.E., Kotha P., Owen H., Patel D., Carter P.W., Cronin J., Green J.-B.D. (2013). Interactions between Parenteral Lipid Emulsions and Container Surfaces. PDA J. Pharm. Sci. Technol..

[34] De Ribeiro D.O., Lobo B.W., Volpato N.M., da Veiga V.F., Cabral L.M., de Sousa V.P. (2009). Influence of the Calcium Concentration in the Presence of Organic Phosphorus on the Physicochemical Compatibility and Stability of All-in-One Admixtures for Neonatal Use. Nutr. J..

[35] Janů M., Brodská H., Vecka M., Masteiková R., Kotrlíková E., Lažauskas R., Pečiūra R., Bernatonienė J. (2011). Comparison of Long-Term Stability of Parenteral All-in-One Admixtures Containing New Lipid Emulsions Prepared under Hospital Pharmacy Conditions. Medicina.

[36] Payne-James J., Grimble G., Silk D., Simon A. (1995). Parenteral Nutrition Formulation. Artificial Nutrition Support in Clinical Practise.

[37] Chaieb D.S., Chaumeil J.C., Jebnoun S., Khrouf N., Hedhili A., Sfar S. (2008). Effect of the Intravenous Lipid Emulsions on the Availability of Calcium When Using Organic Phosphate in TPN Admixtures. Pharm. Res..

[38] CIMA Ficha Técnica Aminoplasmal b. Braun 10% Solución Para Perfusión. https://cima.aemps.es/cima/dochtml/p/67054/P_67054.html.

[39] CIMA Ficha Técnica Aminoven Infant 10% Solución Para Perfusión. https://cima.aemps.es/cima/dochtml/ft/59628/FT_59628.html.

