# Supplementary material for: Physicochemical Stability of Hospital Parenteral Nutrition Solutions: Effect of Changes in Composition and Storage Protocol
Source: Pharmaceutics. 2024 Apr 23;16(5):572. doi: 10.3390/pharmaceutics16050572 (PMC11125120; doi:10.3390/pharmaceutics16050572)
Supplement: Supplementary file 1 [file pharmaceutics-16-00572-s001.zip › pharmaceutics-2958630-supplementary.pdf]

SUPPLEMENTARY MATERIAL:

Table S1. *Calculated droplet diameter parameters for increasing [calcium] samples (PNBASE, PN1-PN4). All days of analysis (1, 3, 10 and 14) and both storage conditions (RT: room temperature, 4ºC: refrigerator).*

Sample	Day	Tª	MDD ± SD (µm)	MDD1 ± SD (µm)	PCT1 (%)	MDD2 ± SD (µm)	PFAT1 ± SD (%)	PFAT2 ± SD (%)
	3	RT	0,437 ± 0	0,416 ± 0	98,54	1,848 ± 0,001	1,458 ± 0,04	0,444 ± 0,01
	10	RT	0,401 ± 0,001	0,401 ± 0,001	100,00	-	-	-
	14	RT	0,437 ± 0	0,417 ± 0	98,66	1,886 ± 0,004	1,344 ± 0,021	0,506 ± 0,006
NPBASE	1	4ºC	0,441 ± 0,002	0,418 ± 0	98,30	1,763 ± 0,083	1,699 ± 0,039	0,286 ± 0,251
	3	4ºC	0,420 ± 0	0,420 ± 0	100,00	-	-	-
	10	4ºC	0,416 ± 0,001	0,416 ± 0,001	100,00	-	-	-
	14	4ºC	0,428 ± 0,001	0,416 ± 0	99,04	1,689 ± 0,014	0,955 ± 0,032	0,047 ± 0,02
	3	RT	0,420 ± 0	0,420 ± 0	100,00	-	-	-
	10	RT	0,417 ± 0,001	0,417 ± 0,001	100,00	-	-	-
	14	RT	0,439 ± 0,002	0,418 ± 0	98,54	1,864 ± 0,024	1,462 ± 0,073	0,49 ± 0,1
PN1	1	4ºC	0,431 ± 0,001	0,417 ± 0	98,92	1,696 ± 0,019	1,078 ± 0,044	0,065 ± 0,023
	3	4ºC	0,422 ± 0	0,422 ± 0	100,00	-	-	-
	10	4ºC	0,417 ± 0,001	0,417 ± 0,001	100,00	-	-	-
	14	4ºC	0,435 ± 0,001	0,417 ± 0	98,62	1,759 ± 0,014	1,384 ± 0,019	0,211 ± 0,037
	3	RT	0,421 ± 0	0,421 ± 0	100,00	-	-	-
	10	RT	0,422 ± 0,001	0,420 ± 0	99,82	1,409 ± 0,027	0,181 ± 0,076	-
	14	RT	0,438 ± 0,002	0,417 ± 0,001	98,53	1,842 ± 0,032	1,469 ± 0,061	0,433 ± 0,115
PN2	1	4ºC	0,432 ± 0,001	0,417 ± 0	98,84	1,723 ± 0,013	1,16 ± 0,043	0,112 ± 0,029
	3	4ºC	0,427 ± 0,001	0,421 ± 0	99,53	1,525 ± 0,009	0,472 ± 0,051	-
	10	4ºC	0,422 ± 0,001	0,422 ± 0,001	100,00	-	-	-
	14	4ºC	0,444 ± 0,001	0,418 ± 0	98,17	1,853 ± 0,008	1,833 ± 0,008	0,59 ± 0,028
	3	RT	0,422 ± 0,001	0,422 ± 0,001	100,00	-	-	-
	10	RT	0,413 ± 0,001	0,413 ± 0,001	100,00	-	-	-
	14	RT	0,429 ± 0,001	0,416 ± 0	99,06	1,758 ± 0,015	0,936 ± 0,064	0,129 ± 0,035
PN3	1	4ºC	0,43 ± 0,001	0,416 ± 0	98,92	1,723 ± 0,012	1,084 ± 0,036	0,099 ± 0,019
	3	4ºC	0,426 ± 0	0,42 ± 0	99,53	1,546 ± 0,014	0,467 ± 0,036	-
	10	4ºC	0,420 ± 0	0,42 ± 0	100,00	-	-	-
	14	4ºC	0,439 ± 0	0,417 ± 0	98,46	1,835 ± 0,006	1,541 ± 0,016	0,437 ± 0,016
	3	RT	0,431 ± 0	0,416 ± 0	98,91	1,81 ± 0,01	1,089 ± 0,019	0,249 ± 0,025
	10	RT	0,422 ± 0,001	0,422 ± 0,001	100,00	-	-	-
	14	RT	0,455 ± 0,001	0,424 ± 0	97,92	1,914 ± 0,003	2,081 ± 0,052	0,898 ± 0,035
PN4	1	4ºC	0,432 ± 0	0,420 ± 0	98,92	1,575 ± 0,002	1,075 ± 0,013	-
	3	4ºC	0,389 ± 0,001	0,389 ± 0,001	100,00	-	-	-
	10	4ºC	0,425 ± 0	0,425 ± 0	100,00	-	-	-
	14	4ºC	0,482 ± 0,001	0,430 ± 0	96,60	1,971 ± 0,02	3,404 ± 0,025	1,755 ± 0,085

Results where the fraction of droplets >1µm is >5% and those exceeding USP specifications are indicated in bold; SD: standard deviation relative to the technical triplicate performed on each day of measurement; Tª: storage temperature; MDD: mean total droplet diameter; TMG1: mean droplet diameter of the first peak; MDD2: mean droplet diameter of the second peak; PCT1: percentage distribution of peak 1 in relation to the total; PFAT1: percentage of droplets larger than 1µm; PFAT2: percentage of droplets larger than 2µm.

Table S2. *Calculated droplet diameter parameters for increasing [magnesium] samples (PN5-PN8). All days of analysis (1, 3, 10 and 14) and both storage conditions (RT: room temperature, 4ºC: refrigerator).*

Sample	Day	Tª	MDD ± SD (µm)	MDD1 ± SD (µm)	PCT1 (%)	MDD2 ± SD (µm)	PFAT1 ± SD (%)	PFAT2 ± SD (%)	PFAT3 ± SD (%)
PN5	3	RT	0,394 ± 0	0,394 ± 0	100,00	-	-	-	-
	10	RT	0,406 ± 0	0,406 ± 0	100,00	-	-	-	-
	14	RT	0,456 ± 0,001	0,424 ± 0	97,66	1,825 ± 0,009	2,337 ± 0,01	0,665 ± 0,037	-
	1	4ºC	0,431 ± 0	0,419 ± 0	98,97	1,582 ± 0,003	1,031 ± 0,011	-	-
	3	4ºC	0,391 ± 0,001	0,391 ± 0,001	100,00	-	-	-	-
	10	4ºC	0,390 ± 0	0,390 ± 0	100,00	-	-	-	-
	14	4ºC	0,455 ± 0,001	0,424 ± 0	97,79	1,832 ± 0,009	2,214 ± 0,033	0,652 ± 0,044	-
PN6	3	RT	0,386 ± 0	0,386 ± 0	100,00	-	-	-	-
	10	RT	0,422 ± 0	0,422 ± 0	100,00	-	-	-	-
	14	RT	0,460 ± 0	0,424 ± 0	97,53	1,897 ± 0,005	2,466 ± 0,023	1,000 ± 0,016	-
	1	4ºC	0,426 ± 0	0,418 ± 0	99,34	1,550 ± 0,014	0,66 ± 0,035	-	-
	3	4ºC	0,378 ± 0,001	0,378 ± 0,001	100,00	-	-	-	-
	10	4ºC	0,418 ± 0,001	0,418 ± 0,001	100,00	-	-	-	-
	14	4ºC	0,455 ± 0,001	0,422 ± 0	97,71	1,863 ± 0,007	2,294 ± 0,01	0,798 ± 0,029	-
PN7	3	RT	0,384 ± 0,001	0,384 ± 0,001	100,00	-	-	-	-
	10	RT	0,423 ± 0,001	0,422 ± 0	99,89	-	0,113 ± 0,075	-	-
	14	RT	0,487 ± 0,001	0,429 ± 0	96,33	2 ± 0,004	3,674 ± 0,059	1,970 ± 0,02	0,032 ± 0,013
	1	4ºC	0,424 ± 0	0,418 ± 0	99,46	1,525 ± 0,006	0,538 ± 0,024	-	-
	3	4ºC	0,406 ± 0,001	0,406 ± 0,001	100,00	-	-	-	-
	10	4ºC	0,426 ± 0	0,426 ± 0	99,94	1,347 ± 0,009	0,064 ± 0,088	-	-
	14	4ºC	0,463 ± 0,001	0,423 ± 0	97,35	1,926 ± 0,002	2,650 ± 0,027	1,198 ± 0,018	-
PN8	3	RT	0,397 ± 0,001	0,397 ± 0,001	100,00	-	-	-	-
	10	RT	**0,506 ± 0,003**	0,426 ± 0	95,54	2,062 ± 0,002	4,463 ± 0,091	2,88 ± 0,077	0,541 ± 0,093
	14	RT	0,483 ± 0,001	0,422 ± 0	96,69	2,165 ± 0,002	3,305 ± 0,038	2,459 ± 0,027	0,331 ± 0,024
	1	4ºC	0,427 ± 0	0,415 ± 0	99,10	1,777 ± 0,015	0,895 ± 0,007	0,143 ± 0,018	-
	3	4ºC	0,391 ± 0,01	0,391 ± 0,01	100,00	-	-	-	-
	10	4ºC	0,477 ± 0,002	0,425 ± 0	96,79	2,029 ± 0,007	3,215 ± 0,076	1,868 ± 0,072	0,044 ± 0,025
	14	4ºC	0,435 ± 0,001	0,415 ± 0	98,67	1,859 ± 0,006	1,328 ± 0,034	0,428 ± 0,025	-

Results where the fraction of cells >1µm is >5% and those exceeding USP specifications are indicated in bold; SD: standard deviation relative to the technical triplicate performed on each day of measurement; Tª: storage temperature; A: ambient; N: refrigerator; MDD: mean total cell diameter; MDD1: mean droplet diameter of the first peak; MDD2: mean droplet diameter of the second peak; PCT1: percentage distribution of peak 1 in relation to the total; PFAT1: percentage of droplets larger than 1µm; PFAT2: percentage of droplets larger than 2µm; PFAT3: percentage of droplets larger than 3µm.

Table S3. *Calculated droplet diameter parameters for increasing [phosphorus] samples (PN9-PN12). All days of analysis (1, 3, 10 and 14) and both storage conditions (RT: room temperature, 4ºC: refrigerator).*

Sample	Day	Tª	MDD ± SD (µm)	MDD1 ± SD (µm)	PCT1 (%)	MDD2 ± SD (µm)	PFAT1 ± SD (%)	PFAT2 ± SD (%)	PFAT3 ± SD (%)
PN9	3	RT	0,387 ± 0,006	0,387 ± 0,006	100,00	-	-	-	-
	10	RT	0,442 ± 0,001	0,419 ± 0	98,41	1,895 ± 0,013	1,588 ± 0,044	0,629 ± 0,059	-
	14	RT	0,435 ± 0	0,415 ± 0	98,66	1,865 ± 0	1,340 ± 0,033	0,448 ± 0,012	-
	1	4ºC	0,427 ± 0,001	0,416 ± 0	99,13	1,753 ± 0,006	0,869 ± 0,047	0,110 ± 0,012	-
	3	4ºC	0,386 ± 0,001	0,386 ± 0,001	100,00	-	-	-	-
	10	4ºC	0,444 ± 0,001	0,419 ± 0	98,30	1,896 ± 0,011	1,701 ± 0,037	0,676 ± 0,052	-
	14	4ºC	0,446 ± 0,001	0,418 ± 0	98,21	1,957 ± 0,004	1,79 ± 0,034	0,907 ± 0,024	-
PN10	3	RT	0,397 ± 0,001	0,397 ± 0,001	100,00	-	-	-	-
	10	RT	0,441 ± 0,001	0,418 ± 0	98,50	1,92 ± 0,011	1,505 ± 0,032	0,667 ± 0,045	-
	14	RT	0,437 ± 0,001	0,417 ± 0	98,65	1,932 ± 0,004	1,345 ± 0,025	0,623 ± 0,02	-
	1	4ºC	0,429 ± 0,001	0,416 ± 0	99,05	1,764 ± 0,006	0,953 ± 0,039	0,139 ± 0,016	-
	3	4ºC	0,396 ± 0,001	0,396 ± 0,001	100,00	-	-	-	-
	10	4ºC	0,440 ± 0,001	0,417 ± 0	98,40	1,856 ± 0,019	1,598 ± 0,048	0,513 ± 0,077	-
	14	4ºC	0,416 ± 0	0,411 ± 0	99,61	1,835 ± 0,001	0,39 ± 0,018	0,091 ± 0,006	-
PN11	3	RT	0,394 ± 0,001	0,394 ± 0,001	100,00	-	-	-	-
	10	RT	0,446 ± 0,001	0,42 ± 0	98,25	1,902 ± 0,014	1,755 ± 0,046	0,721 ± 0,067	-
	14	RT	0,441 ± 0,001	0,417 ± 0	98,42	1,93 ± 0,009	1,578 ± 0,029	0,726 ± 0,038	-
	1	4ºC	0,431 ± 0,001	0,416 ± 0	98,92	1,747 ± 0,008	1,079 ± 0,044	0,138 ± 0,017	-
	3	4ºC	0,387 ± 0,001	0,387 ± 0,001	100,00	-	-	-	-
	10	4ºC	0,446 ± 0,001	0,418 ± 0	98,14	1,926 ± 0,013	1,865 ± 0,032	0,850 ± 0,055	-
	14	4ºC	0,454 ± 0,001	0,42 ± 0	97,83	1,998 ± 0,017	2,174 ± 0,044	1,232 ± 0,075	-
PN12	3	RT	0,486 ± 0,001	0,424 ± 0	96,35	2,086 ± 0,003	3,652 ± 0,023	2,374 ± 0,029	0,088 ± 0,022
	10	RT	0,494 ± 0,001	0,422 ± 0	95,81	2,05 ± 0,003	4,187 ± 0,083	2,528 ± 0,059	0,383 ± 0,013
	14	RT	0,437 ± 0,001	0,415 ± 0	98,51	1,876 ± 0,014	1,492 ± 0,037	0,535 ± 0,052	-
	1	4ºC	0,443 ± 0	0,419 ± 0	98,30	1,852 ± 0,006	1,701 ± 0,025	0,541 ± 0,012	-
	3	4ºC	0,47 ± 0,001	0,425 ± 0	97,05	1,934 ± 0,006	2,946 ± 0,018	1,364 ± 0,035	-
	10	4ºC	0,445 ± 0	0,421 ± 0	98,13	1,694 ± 0,006	1,87 ± 0,019	0,179 ± 0,013	-
	14	4ºC	0,453 ± 0,002	0,416 ± 0	97,76	2,047 ± 0,02	2,239 ± 0,079	1,440 ± 0,107	-

Results where the fraction of cells >1µm is >5% and those exceeding USP specifications are indicated in bold; SD: standard deviation relative to the technical triplicate performed on each day of measurement; Tª: storage temperature; A: ambient; N: refrigerator; MDD: mean total cell diameter; MDD1: mean droplet diameter of the first peak; MDD2: mean droplet diameter of the second peak; PCT1: percentage distribution of peak 1 in relation to the total; PFAT1: percentage of droplets larger than 1µm; PFAT2: percentage of droplets larger than 2µm; PFAT3: percentage of droplets larger than 3µm.

Table S4: *Calculated droplet diameter parameters for decreasing [lipid] samples (PN13-PN17). All days of analysis (1, 3, 10 and 14) and both storage conditions (RT: room temperature, 4ºC: refrigerator).*

Sample	Day	Tª	MDD ± SD (µm)	MDD1 ± SD (µm)	PCT1 (%)	MDD2 ± SD (µm)	*MDD3 ± DS (µm)*	PFAT1 ± SD (%)	PFAT2 ± SD (%)	PFAT3 ± SD (%)
PN13	3	RT	0,444 ± 0	0,421 ± 0	98,34	1,841 ± 0,006	-	1,657 ± 0,012	0,504 ± 0,02	-
	10	RT	0,432 ± 0,001	0,418 ± 0	99,01	1,705 ± 0,015	-	1,075 ± 0,093	0,121 ± 0,012	-
	14	RT	0,442 ± 0,002	0,418 ± 0	98,30	1,838 ± 0,021	-	1,700 ± 0,109	0,499 ± 0,088	-
	1	4ºC	0,448 ± 0,003	0,421 ± 0,001	98,11	1,851 ± 0,039	-	1,889 ± 0,076	0,608 ± 0,156	-
	3	4ºC	0,457 ± 0,001	0,426 ± 0	97,51	1,691 ± 0,012	-	2,494 ± 0,012	0,265 ± 0,041	-
	10	4ºC	0,429 ± 0,001	0,423 ± 0,002	99,36	1,330 ± 0,03	-	0,643 ± 0,29	-	-
	14	4ºC	0,435 ± 0,001	0,416 ± 0	98,58	1,749 ± 0,011	-	1,422 ± 0,04	0,199 ± 0,026	-
PN14	3	RT	0,425 ± 0	0,421 ± 0	99,68	1,528 ± 0,005	-	0,318 ± 0,008	-	-
	10	RT	0,422 ± 0,001	0,419 ± 0	99,73	-	-	0,271 ± 0,138	-	-
	14	RT	0,456 ± 0,002	0,422 ± 0	97,72	1,894 ± 0,018	-	2,285 ± 0,102	0,913 ± 0,104	-
	1	4ºC	0,478 ± 0,004	0,426 ± 0	96,68	1,985 ± 0,006	-	3,324 ± 0,171	1,729 ± 0,128	0,021 ± 0,073
	3	4ºC	0,456 ± 0,001	0,425 ± 0,001	97,47	1,668 ± 0,014	-	2,527 ± 0,025	0,208 ± 0,036	-
	10	4ºC	0,435 ± 0,001	0,429 ± 0,002	99,28	1,256 ± 0,027	-	0,720 ± 0,338	-	-
	14	4ºC	0,448 ± 0,002	0,420 ± 0	97,95	1,803 ± 0,029	-	2,050 ± 0,128	0,500 ± 0,109	-
PN15	3	RT	**0,514 ± 0,005**	0,423 ± 0	95,92	2,006 ± 0,009	3,854 ± 0,102	4,075 ± 0,11	2,955 ± 0,123	1,466 ± 0,086
	10	RT	0,409 ± 0,003	0,409 ± 0,003	100,00	-	-	-	-	-
	14	RT	0,451 ± 0,004	0,417 ± 0	97,96	2,052 ± 0,028	-	2,044 ± 0,186	1,326 ± 0,184	-
	1	4ºC	0,474 ± 0,001	0,424 ± 0	96,81	2,002 ± 0,01	-	3,191 ± 0,054	1,763 ± 0,063	-
	3	4ºC	**0,503 ± 0,013**	0,431 ± 0,002	95,73	1,959 ± 0,006	3,386 ± 0,106	4,273 ± 0,41	2,277 ± 0,478	0,460 ± 0,518
	10	4ºC	0,44 ± 0,004	0,419 ± 0	98,25	1,614 ± 0,062	-	1,755 ± 0,274	0,044 ± 0,025	-
	14	4ºC	0,45 ± 0,003	0,419 ± 0	97,92	1,924 ± 0,026	-	2,076 ± 0,138	0,929 ± 0,143	-
PN16	3	RT	**0,562 ± 0,014**	0,435 ± 0,002	**91,68**	1,913 ± 0,012	2,888 ± 0	8,317 ± 0,876	3,574 ± 0,35	0,366 ± 0,046
	10	RT	**0,593 ± 0,009**	0,419 ± 0,001	**92,73**	2,073 ± 0,01	3,736 ± 0,076	7,275 ± 0,285	5,603 ± 0,23	3,113 ± 0,094
	14	RT	**0,509 ± 0,019**	0,419 ± 0	95,76	2,119 ± 0,004	3,362 ± 0,343	4,241 ± 0,392	3,238 ± 0,442	1,363 ± 0,421
	1	4ºC	**0,579 ± 0,029**	0,449 ± 0,007	**89,68**	1,708 ± 0,071	-	10,324 ± 1,359	2,383 ± 1,059	-
	3	4ºC	**0,554 ± 0,029**	0,432 ± 0,004	**92,92**	1,918 ± 0,039	3,151 ± 0,095	7,078 ± 1,282	3,555 ± 0,66	1,307 ± 0,543
	10	4ºC	**0,52 ± 0,005**	0,422 ± 0	**94,85**	1,992 ± 0,002	3,281 ± 0,002	5,153 ± 0,201	3,175 ± 0,139	1,248 ± 0,085
	14	4ºC	0,387 ± 0	0,387 ± 0	100,00	-	-	-	-	-
PN17	3	RT	**0,617 ± 0,005**	0,435 ± 0,001	**87,57**	1,891 ± 0,013	2,824 ± 0,036	12,434 ± 0,099	5,838 ± 0,067	-
	10	RT	**0,561 ± 0,001**	0,430 ± 0	**92,08**	2,083 ± 0,002	-	7,920 ± 0,032	5,319 ± 0,04	-
	14	RT	0,383 ± 0,004	0,383 ± 0,004	100,00	-	-	-	-	-
	1	4ºC	**0,734 ± 0,004**	0,460 ± 0,002	**78,76**	1,714 ± 0,001	2,885 ± 0	21,245 ± 0,184	6,598 ± 0,117	0,453 ± 0,015
	3	4ºC	**0,594 ± 0,001**	0,433 ± 0	**90,44**	1,977 ± 0,002	2,962 ± 0	9,563 ± 0,034	4,908 ± 0,031	1,246 ± 0,01
	10	4ºC	**0,502 ± 0,002**	0,427 ± 0	95,17	1,988 ± 0,01	-	4,830 ± 0,197	2,535 ± 0,048	-
	14	4ºC	0,394 ± 0	0,394 ± 0	100,00	-	-	-	-	-

Results where the fraction of droplets >1µm is >5% and those exceeding USP specifications are indicated in bold; SD: standard deviation relative to the technical triplicate performed on each day of measurement; Tª: storage temperature; A: ambient; N: refrigerator; MDD: mean total droplet diameter; MDD1: mean droplet diameter of the first peak; MDD2: mean droplet diameter of the second peak; MDD3: mean droplet diameter of the third peak; PCT1: percentage distribution of peak 1 out of the total; PFAT1: percentage of droplets larger than 1µm; PFAT2: percentage of droplets larger than 2µm; PFAT3: percentage of droplets larger than 3µm.

Table S5: *Calculated droplet diameter parameters for decreasing [amino acid] samples (PN18-PN22). All days of analysis (1, 3, 10 and 14) and both storage conditions (RT: room temperature, 4ºC: refrigerator).*

Sample	Day	Tª	MDD ± SD (µm)	MDD1 ± SD (µm)	PCT1 (%)	MDD2 ± SD (µm)	*MDD3 ± DS (µm)*	*PFAT1 ± DS (%)*	*PFAT3 ± DS (%)*	*PFAT5 ± DS (%)*
	3	RT	0,499 ± 0,003	0,429 ± 0	95,37	1,873 ± 0,007	3,052 ± 0,05	4,632 ± 0,095	0,221 ± 0,051	-
	10	RT	0,451 ± 0	0,416 ± 0	97,68	1,918 ± 0,007	-	2,324 ± 0,021	-	-
	14	RT	0,387 ± 0	0,387 ± 0	100,00	-	-	-	-	-
PN18	1	4ºC	**0,579 ± 0,006**	0,443 ± 0	**89,66**	1,761 ± 0,017	-	10,335 ± 0,307	0,005 ± 0,003	-
	3	4ºC	0,505 ± 0	0,432 ± 0	95,09	1,879 ± 0,004	2,958 ± 0	4,911 ± 0,029	0,153 ± 0,002	-
	10	4ºC	0,448 ± 0,001	0,416 ± 0	97,80	1,879 ± 0,002	-	2,198 ± 0,027	-	-
	14	4ºC	0,393 ± 0	0,393 ± 0	100,00	-	-	-	-	-
	3	RT	0,499 ± 0,003	0,429 ± 0	95,37	1,873 ± 0,007	3,052 ± 0,05	4,632 ± 0,095	0,221 ± 0,051	-
	10	RT	**0,503 ± 0,001**	0,422 ± 0	95,43	2,007 ± 0,005	3,247 ± 0,006	4,572 ± 0,041	0,641 ± 0,028	-
	14	RT	0,402 ± 0	0,402 ± 0	100,00	-	-	-	-	-
PN19	1	4ºC	**0,576 ± 0,011**	0,440 ± 0,002	**89,90**	1,785 ± 0,037	-	10,095 ± 0,411	-	-
	3	4ºC	0,497 ± 0	0,430 ± 0	95,39	1,874 ± 0	2,957 ± 0,032	4,608 ± 0,047	0,096 ± 0,01	-
	10	4ºC	0,463 ± 0	0,419 ± 0	97,12	1,949 ± 0,002	-	2,881 ± 0,031	-	-
	14	4ºC	0,400 ± 0	0,400 ± 0	100,00	-	-	-	-	-
	3	RT	0,395 ± 0	0,395 ± 0	100,00	-	-	-	-	-
	10	RT	0,438 ± 0,001	0,413 ± 0	98,38	1,99 ± 0,006	-	1,619 ± 0,018	-	-
	14	RT	0,491 ± 0	0,416 ± 0	95,77	2,158 ± 0,003	2,935 ± 0,002	4,228 ± 0,018	0,167 ± 0,006	-
PN20	1	4ºC	0,437 ± 0	0,414 ± 0	98,54	1,935 ± 0,013	-	1,462 ± 0,02	-	-
	3	4ºC	0,431 ± 0	0,413 ± 0	98,69	1,765 ± 0,007	-	1,307 ± 0,027	-	-
	10	4ºC	0,433 ± 0	0,413 ± 0	98,58	1,839 ± 0,002	-	1,419 ± 0,005	-	-
	14	4ºC	0,448 ± 0,002	0,413 ± 0,001	97,98	2,159 ± 0,038	2,824 ± 0	2,020 ± 0,071	-	-
	3	RT	0,410 ± 0	0,410 ± 0	100,00	-	-	-	-	-
	10	RT	**0,508 ± 0,003**	0,416 ± 0	95,75	2,163 ± 0,009	3,319 ± 0,005	4,252 ± 0,098	1,404 ± 0,141	-
	14	RT	**0,847 ± 0,017**	0,424 ± 0	**84,52**	2,129 ± 0,004	3,815 ± 0,137	15,481 ± 0,139	9,068 ± 0,111	**0,746 ± 0,139**
PN21	1	4ºC	0,412 ± 0	0,412 ± 0	100,00	-	-	-	-	-
	3	4ºC	0,406 ± 0	0,406 ± 0	100,00	-	-	-	-	-
	10	4ºC	0,445 ± 0	0,416 ± 0	98,05	1,889 ± 0,003	-	1,947 ± 0,014	-	-
	14	4ºC	0,441 ± 0,001	0,412 ± 0	98,20	2,012 ± 0,007	-	1,799 ± 0,023	-	-
	3	RT	**0,523 ± 0,001**	0,422 ± 0	**94,32**	1,945 ± 0,003	3,158 ± 0,002	5,682 ± 0,037	1,204 ± 0,048	-
	10	RT	**0,688 ± 0,008**	0,428 ± 0	**88,92**	2,018 ± 0,004	3,960 ± 0,067	11,082 ± 0,124	4,124 ± 0,133	**0,736 ± 0,124**
	14	RT	**0,773 ± 0,016**	0,436 ± 0,001	**84,00**	2,017 ± 0,001	3,579 ± 0,1	16,004 ± 0,308	5,078 ± 0,333	**0,13 ± 0,308**
PN22	1	4ºC	0,437 ± 0	0,413 ± 0	98,47	1,939 ± 0,012	-	1,527 ± 0,018	-	-
	3	4ºC	0,495 ± 0,003	0,420 ± 0	95,57	1,961 ± 0,01	3,061 ± 0,062	4,427 ± 0,086	0,617 ± 0,136	-
	10	4ºC	**0,646 ± 0,011**	0,428 ± 0	**89,92**	2,011 ± 0,006	3,656 ± 0,068	10,076 ± 0,228	3,404 ± 0,234	-
	14	4ºC	**0,635 ± 0,012**	0,423 ± 0	**90,73**	2,055 ± 0,004	3,735 ± 0,076	9,272 ± 0,262	3,453 ± 0,252	**0,117 ± 0,262**

Results where the fraction of droplets >1µm is >5% and those exceeding USP specifications are indicated in bold; SD: standard deviation relative to the technical triplicate performed on each day of measurement; Tª: storage temperature; A: ambient; N: refrigerator; TMGt: mean total droplet diameter; MDD1: mean droplet diameter of the first peak; MDD2: mean droplet diameter of the second peak; MDD3: mean droplet diameter of the third peak; PCT1: percentage distribution of peak 1 out of the total; PFAT1: percentage of droplets larger than 1µm; PFAT3: percentage of droplets larger than 3µm; PFAT5: percentage of droplets larger than 5µm.
